# Obesity is always a clinically relevant chronic disease

**DOI:** 10.1007/s40519-026-01820-0

**Published:** 2026-04-18

**Authors:** Lorenzo M. Donini, Maria D. Ballesteros-Pomar, John A. Batsis, Yves Boirie, Gianluca Gortan Cappellari, Eleonora Poggiogalle, Rocco Barazzoni

**Affiliations:** 1 Department of Experimental Medicine, Sapienza University, Rome, Italy; 2 Department of Endocrinology and Nutrition, Complejo Asistencial Universitario de León, Leon, Spain; 3 Division of Geriatric Medicine, School of Medicine, Department of Nutrition, The Gillings School of Global Public Health, Chapel Hill, NC USA; 4 Clinical Nutrition Department, University Hospital of Clermont-Ferrand, Clermont-Ferrand, France; 5 Human Nutrition Unit, INRAe, Centre de Recherche en Nutrition Humaine, Clermont Auvergne University, Clermont-Ferrand, France; 6 Department of Medical, Surgical and Health Sciences, University of Trieste, Trieste, Italy

**Keywords:** Obesity, Chronic disease, Obesity medication, Bio-psycho-social model

## Abstract

Interest in obesity has grown exponentially over the last years, with the availability of highly effective new pharmacological treatment options. The increasing use of pharmacological treatment options has stimulated debates on several fundamental issues, including (1) full recognition of obesity as a disease, and (2) optimization of the diagnostic criteria of obesity and the timing for offering different treatment options. We aim at critically discussing here the similarities, discrepancies, and potential misunderstandings suggested by the European Association for the Study of Obesity (EASO) and Lancet Diabetes Endocrinology Commission statements. In particular, two aspects are discussed: (1) the BMI limitations and the necessity to include visceral fat and, more in general, body composition assessment in the diagnosis of obesity; (2) the opportunity to consider obesity always as a clinically relevant chronic disease due to its biological, psychological, and social characteristics.

Interest in obesity has grown exponentially over the last years, with the availability of highly effective new pharmacological treatment options [1, 2]. The increasing use of pharmacological treatment options has stimulated debates on several fundamental issues, including (1) full recognition of obesity as a disease, and more specifically a chronic, progressive relapsing one, as introduced by the World Health Organization [3] and (2) optimization of the diagnostic criteria of obesity and the timing for offering different treatment options. Among other initiatives, the European Association for the Study of Obesity (EASO) has published documents on both diagnostic and treatment conceptual frameworks [4, 5]. A large international group of > 50 experts has also been invited by the Lancet to discuss these topics, resulting in a comprehensive paper including guidance on obesity diagnostic assessment and treatment approaches [6]. We aim at critically discussing here the similarities, discrepancies, and potential misunderstandings suggested by the EASO and Lancet Commission statements.

## Obesity diagnosis: BMI limitations

1. *Visceral fat in obesity diagnosis*: Limitations of body mass index (BMI) in identifying not only obesity-associated clinical risk, but also different clinically relevant obesity phenotypes have been long acknowledged. WHO defines obesity as a disease characterized by excessive fat accumulation, which has been translated into the concept of “adiposity-based chronic disease” (ABCD) by the American Association of Clinical Endocrinologists [7] and the EASO [8]. A high BMI is an invaluable tool to identify excess body mass as a proxy for excess body fat in clinical practice, and it remains a cornerstone for clinical obesity diagnosis and monitoring. However, BMI does not provide information on either fat amount or its distribution in the body and in the organs. Abdominal visceral fat accumulation can be detected using simple anthropometric surrogate measures based on waist circumference (WC) [9], and all recent statements have strongly recommended the inclusion of WC, waist-to-hip, or waist-to-height in the diagnostic algorithm for obesity (LC). In particular, EASO focuses on waist-to-height ratio (EASO) with consideration of gender and ethnicity-specific cut-offs. The EASO statement has further proposed to consider elevated waist-to-height ratio as a diagnostic criterion for obesity also in the presence of BMI levels that were previously considered in the overweight range (BMI > 25 kg/m^2^) [4]. Similarly, the Lancet Commission defined WC-based measurements as “body size measurements” and proposed to confirm obesity diagnosis in the presence of at least two out of three altered parameters, regardless of elevated BMI [6]. Thus, both statements move towards increasing consideration for abdominal visceral fat in obesity diagnosis, based on its predictive role for elevated health risk and complications [10, 11]. These combined messages should hopefully strengthen awareness on the fundamental role of abdominal visceral fat in the pathophysiology and clinical history of obesity.
2. *Body composition (beyond body fat)*:While the importance of abdominal visceral fat has been long recognized, the general concept of altered body composition and its profound clinical impact in persons with obesity is less appreciated, and they remain under-discussed in the EASO and Lancet Commission statements. The Lancet Commission included elevated body fat as an independent diagnostic criterion, and indeed sufficient for diagnosing obesity; although, no threshold for diagnosis was provided. It should also be pointed out that excess fat accumulation, per se, is inherently coupled to relative or absolute reductions in non-fat body compartments [12] that may pose independent clinical challenges. Low muscle mass relative to total body weight, coupled with low muscle strength, has been recently introduced as a diagnostic criterion for sarcopenic obesity, and its negative clinical impact has been confirmed in recent years [13, 14]. While an in-depth discussion of sarcopenic obesity is beyond the goals of the current paper, comprehensive assessment of body composition in clinical practice should be an important and timely discussion point for advocacy in the obesity community. This is all the more relevant at a time of rapid development in therapeutics for obesity management, with the introduction of obesity medications that likely have a profound impact on body composition [15, 16] and whose risk–benefit assessment should become part of routine evaluation when the risk of substantial muscle loss is elevated [17]. The EASO statement indeed supports the assessment of both muscle parameters and sarcopenic obesity, particularly among older adults [4]. Besides and beyond more accurate measurement of excess body fat and its distribution, we strongly advocate for further implementation of body composition assessment in clinical practice, with priority for all patient groups at higher risk for loss of muscle mass and function [13], using available techniques such as bioimpedance analysis (BIA) or dual-energy X-ray absorptiometry (DXA). We further advocate for clinical and technological research to improve technical accuracy coupled with practical availability.

## Obesity as a disease—clinical impact and treatment complications

*Why obesity is a disease*: obesity has been recognized as a chronic disease by clinicians and policymakers in a number of statements and settings [18–21]. However, this concept is not yet accepted in public awareness. Most importantly, patients and, to some extent, healthcare professionals, particularly those not involved in obesity care, still more or less openly fail to coherently embrace the concept that obesity is a chronic, progressive, and relapsing disease. On the contrary, obesity is unfortunately often considered a condition resulting from wrong choices and personal responsibilities in adopting “wrong” obesogenic lifestyles and not having the willingness or the strength to modify them. This judgemental approach is wrong and dangerous, leading to the obesity stigma that affects not only patients living with obesity but also the whole field of obesity, making allocation of resources for effective treatment extremely difficult. The body dissatisfaction and the obesity stigma favour thinness and criticize fat bodies, leading to “fatphobia” and the spread of eating disorders [22].

We only wish to summarize below the main reasons why obesity fulfils all criteria for the definition of chronic disease, within the most accepted WHO-endorsed bio-psycho-social model, requiring biomedical, psychological, and social components. Further details are provided in recent reviews [23, 24]. In summary:

- *Biomedical component*: biological mechanisms regulating energy balance, including appetite regulation and energy expenditure, are altered in obesity in pre-clinical and clinical studies [25]. For instance, derangements include inefficient homeostatic and reward-associated components of appetite regulation, and altered energy expenditure through lipid oxidation and thermogenesis [26, 27]. Also relevant, excess adipose tissue may also undergo maladaptive changes and fibrosis that make it a source of systemic inflammation with further negative impact on appetite regulation, reflecting the ABCD model [28] with a strong pathogenetic role in the development of cardio-metabolic and other systemic complications [29].
- *Psychological component*: multiple psychological disorders contribute to the onset and complications of obesity. Data from the literature suggest a high prevalence of feeding and eating disorders in subjects with obesity: up to 79% present binge-eating symptoms and up to 25% night eating syndrome in the different studies [30]. Beyond impaired reward mechanisms and their direct contribution to positive energy balance, a subgroup of patients demonstrates impulsive/compulsive eating behaviours (binge eating). These primary components also play a role in the commonly observed development of anxiety and depressive symptoms, with altered response to stress, which leads to further behavioural modifications.
- *Social component*: obesity is strongly influenced by socioeconomic and environmental factors in many ways. High food availability, lower food quality, and substantially reduced opportunities for physical activity and exercise are the main components of the obesogenic environment, which have interacted with biological and exposome-based factors, allowing the development of the ongoing global obesity epidemic. Social inequalities (e.g. disparities in access to health care and job opportunities) also exist in exposure to the risk of developing obesity, with lower socio-cultural level representing a strong risk factor worldwide.
- *Exposome component*: according to the exposome hypothesis, obesity arises from the complex interplay between genetic predisposition and the sum of all environmental exposures (such as diet, social environment, chemicals, stress, infections, and other external and internal factors) throughout a person’s life. This hypothesis highlights that obesity not only results from lifestyle or genetics alone, but also from the cumulative lifelong influence of a wide range of exposures [28].

We therefore maintain that obesity must be considered a chronic disease (which needs long-term monitoring with a systemic approach), defined by altered body composition through excess body fat accumulation [29]. Most importantly, in a large proportion of affected individuals, the onset of the disease is associated with stigma-associated distress and suffering, psychological distress, reduced quality of life, gradual limitations in routine activities as well as triggering of adiposity-based metabolic derangements that lead to systemic complications. Obesity, conversely, represents a major risk factor for a number of systemic complications, including all major non-communicable diseases as well as their acute complications, frailty, disabilities, and mental diseases.

*Lancet commission proposal: pre-clinical and clinical obesity*: the Lancet Commission document [6] has proposed a new classification into pre-clinical and clinical obesity. Interestingly, clinical obesity was defined as “a chronic disease …. characterised by signs and symptoms of ongoing organ dysfunction and/or reduced ability to conduct daily activities”, with a list of specific criteria to define organ dysfunction and autonomy levels that generally require the presence of symptomatic complications. On the other hand, pre-clinical obesity was defined as a “condition of excess body fat associated with variable level of health risk, but no ongoing illness” which “does not represent a disease diagnosis per se”. Importantly, pre-clinical obesity is not therefore proposed to represent an early disease stage, in the general absence of a staging framework.

–*Implications for obesity treatment*: somewhat consistent with the above concepts, the Lancet Commission proposal recommends differential approaches for managing pre-clinical and clinical obesity. For the latter, focusing on “improvement or reversal of organ dysfunction” is recommended, as assessed by improvement of signs and symptoms rather than measures of weight loss. It is said that “people should have access to comprehensive care and evidence-based treatments to induce improvement (or remission when possible) of clinical manifestations of obesity and to prevent progression to end-organ damage developing clinical obesity as well as several other non-communicable diseases (NCDs), including type 2 diabetes, cardiovascular disease, certain types of cancer and mental illness, among others”.

For pre-clinical obesity, focusing on risk reduction and prevention of progression is recommended through health counselling for weight loss or prevention of weight gain, and monitoring over time. It should be also pointed out that “level of care and type of intervention …. should be based on individual risk/benefit assessment, considering the severity of excess/abnormal adiposity and the presence/absence of other risk factors”.

The overall approach proposed by the Lancet Commission raises the following concerns (Table 1):

- It denies the defining obesity derangement (excess fat accumulation) as a disease in its own right; this indeed denies the above-mentioned criteria for disease definition and poses a high risk of perpetuating the obesity stigma for patients as well as a lack of resources for obesity management at large.
- It does not consider that comprehensive lifestyle interventions are effective only for a limited number of persons with obesity, suggesting that the patient is responsible for the prevention of “clinical obesity” [31–33]. This amplifies the bias and stigma against persons with obesity who should be able to manage obesity with lifestyle approaches (somehow going back to 'eat less, move more').
- It separates the disease from its complications, indeed defining the disease a “condition” until complications are clinically evident. This concept is at odds with a number of diseases where a continuum is accepted between the onset of the defining derangement and progressive development of complications (e.g. diabetes, hypertension, organ failures). No one would dispute the need to treat diabetes without waiting for complications to arise.
- It appears to limit the concept of active early treatment for prevention of disease progression and complications by indicating health counselling as the primary intervention for pre-clinical obesity management. Early treatment has been long recognized as a priority for diabetes, hypertension, dyslipidemia, or any other disease whose defining derangement (high blood glucose, high blood pressure, low haemoglobin) can be completely asymptomatic but is nevertheless actively managed in order to prevent complications or organ damage in the mid- to long-term. In obesity, limiting the concept of early treatment and prevention of complications is particularly alarming for children and adolescents that are exposed to a longer duration of fat accumulation and may experience earlier onset of life-threatening complications.
- It proposes a somewhat conservative approach for the detection of complications for clinical obesity, based on “medical history, physical examination and standard blood tests”, including questions addressing “signs/symptoms of organ dysfunction” or “limitations of day-to-day activities”. Clinically advanced conditions are explicitly listed for the diagnosis of clinical obesity, such as the presence of NAFLD with liver fibrosis, obstructive sleep apnoea syndrome, or fatigue and lower limb oedema for heart failure. Such manifestations are characterized by poorer prognosis and could be prevented by actively managing obesity in earlier, “pre-clinical” stages.
- It appears to exclude obesity treatment itself from first-line management of clinical obesity, which rather focuses on specific organ dysfunction. Although diabetes management may be a notable exception, since obesity treatment is indicated in type 2 diabetes, the statement is generally at odds with growing evidence that obesity treatment improves or reverses several major complications.

**Table 1:** Concerns raised by the classification into “preclinical” and “clinical” obesity proposed by the Lancet Commission

It denies the defining obesity derangement (excess fat accumulation) as a disease in its own right	
It does not consider that comprehensive lifestyle interventions are effective only for a limited number of persons with obesity, suggesting that the patient is responsible for the prevention of “clinical obesity”	
It separates the disease from its complications, indeed defining the disease as a “condition” until complications are clinically evident	
It appears to limit the concept of active early treatment for prevention of disease progression and complications, by indicating health counselling as the primary intervention for pre-clinical obesity management	
It proposes a somewhat conservative approach for the detection of complications for clinical obesity, based on “medical history, physical examination and standard blood tests”, including	
It appears to exclude obesity treatment itself from first-line management of clinical obesity, which rather focuses on specific organ dysfunction	

These concerns have been addressed by EASO concerning in particular the anthropometric and clinical components for obesity diagnosis, staging and management: in 2024 and 2025 the European Association for the Study of Obesity has published two papers aimed at providing a concept [4] and a framework focusing specifically on pharmacological treatment of obesity and its complications (2025) [5]:

- *Obesity diagnosis*: the initial framework proposes a comprehensive approach to obesity diagnosis, whereas its anthropometric component (as described above, with emphasized abdominal fat identification) should be associated with the assessment of complications in the medical, functional, and mental domains. Clinical assessment was not described in detail, although references were provided to different available guidelines and guidance documents.
- *Obesity staging*: the same framework provided a conceptual recommendation that obesity be staged after diagnosis based on the presence and severity of its clinical component. The validity of the staging criterion, although the result of a consensus of experts from EASO, is currently being verified. In fact, different approaches for the staging of obesity, based on phenotyping and pathophysiology [34], on the combination of organic, mental, and functional aspects [35], considering the severity of the disease [36], may be proposed to define a different combination/intensity of interventions. Considering obesity as a continuum, with different stages of severity, better approximates the similar context of other metabolic diseases than considering it in mutually exclusive preclinical/clinical categories.
- *Management*: a multimodal approach for obesity management was proposed, with comments on limited data availability on pharmacological and surgical treatment beyond BMI-based categories. In the context of a comprehensive clinical evaluation and with strong clinical emphasis on abdominal fat excess, the paper advocated for more research and clinical trials for pharmacological treatment focusing on these components. Targets for obesity management should emphasize improvement of its clinical component, beyond weight management. The framework on pharmacological treatment of obesity [5] further reinforced this concept, while further emphasizing the indication for pharmacological treatment for weight management in the absence of complications, stating “total weight loss remains an important primary goal of treatment to reduce the risk of incident complications”.

## Conclusions

Rapid evolution in obesity management opportunities has stimulated debate not only on optimization of obesity treatment, but also on its diagnostic components and its nature as a chronic disease leading to potentially fatal systemic complications. The EASO and Lancet Commission statements provide relevant contributions to this discussion. Expanding diagnosis to integrate abdominal visceral fat surrogates is not controversial, although consensus on anthropometric surrogates for abdominal visceral fat and their integration with altered BMI levels is still lacking. There is also emerging agreement on the need to integrate obesity complications and clinical domains beyond excess body fat accumulation, although proposed algorithms present significant conceptual differences. Perhaps unexpectedly, the Lancet Commission document has raised uncertainties and could cause potential confusion on the well-established nature of obesity as a chronic disease defined by pathological fat accumulation PER SE, independent of the presence of clinical complications. As discussed above, the proposal of pre-clinical obesity as an entity where excess fat is (1) not considered a disease per se and (2) is not considered a relevant health problem in the absence of advanced, clinically relevant complications raises significant concerns. This conceptual framework has the potential to lead to major setbacks in the fight against obesity stigma, the call for adequate resourcing to manage obesity, in reinforcing the need for early treatment of excess fat accumulation, to prevent complications, but also for immediate relief of its independent negative impact on health and health status. On a positive note, the Lancet Commission stated that “for some people with pre-clinical obesity and higher overall health risk, other interventions (pharmacological or surgical) might be warranted, proportional to the level of risk and the presence of other conditions that could benefit from reduction of weight or adiposity”. A better definition of indications for more aggressive management could help reduce conceptual and practical discrepancies and concerns. We have ongoing concerns regarding the negative consequences of a simplistic interpretation that managing pre-clinical obesity should normally be limited to lifestyle modification, by providing advice, and in clinicians monitoring the patient’s general health risk.

## Data Availability

No datasets were generated or analysed during the current study.
